# Prevalence and Course of IgA and IgG Antibodies against SARS-CoV-2 in Healthcare Workers during the First Wave of the COVID-19 Outbreak in Germany: Interim Results from an Ongoing Observational Cohort Study

**DOI:** 10.3390/healthcare9050498

**Published:** 2021-04-22

**Authors:** Mark Reinwald, Peter Markus Deckert, Oliver Ritter, Henrike Andresen, Andreas G. Schreyer, Karsten Henrich Weylandt, Werner Dammermann, Stefan Lüth

**Affiliations:** 1 Department of Hematology and Oncology, Brandenburg Medical School Theodor Fontane, 14770 Brandenburg an der Havel, Germany; deckert@klinikum-brandenburg.de; 2 Department of Cardiology and Pneumology, Brandenburg Medical School Theodor Fontane, 14770 Brandenburg an der Havel, Germany; o.ritter@klinikum-brandenburg.de (O.R.); h.andresen@klinikum-brandenburg.de (H.A.); 3 Department of Radiology, Brandenburg Medical School Theodor Fontane, 14770 Brandenburg an der Havel, Germany; a.schreyer@klinikum-brandenburg.de; 4 Department of Gastroenterology, Metabolism and Oncology, Brandenburg Medical School Theodor Fontane, 16816 Neuruppin, Germany; karsten.weylandt@mhb-fontane.de; 5 Faculty of Health Sciences Brandenburg, Brandenburg Medical School Theodor Fontane, 14770 Brandenburg an der Havel, Germany; w.dammermann@klinikum-brandenburg.de (W.D.); s.lueth@klinikum-brandenburg.de (S.L.); 6 Department of Gastroenterology, Brandenburg Medical School Theodor Fontane, 14770 Brandenburg an der Havel, Germany

**Keywords:** SARS-CoV-2, healthcare workers, seroprevalence, COVID-19, anti-SARS-CoV-2 IgA, symptoms, IgG response, first wave, Germany

## Abstract

(1) Background: Healthcare workers (HCWs) are prone to intensified exposure to severe acute respiratory syndrome coronavirus-2 (SARS-CoV-2) infection in the ongoing pandemic. We prospectively analyzed the prevalence of antibodies against SARS-CoV-2 in HCWs at baseline and follow up with regard to clinical signs and symptoms in two university hospitals in Brandenburg, Germany. (2) Methods: Screening for anti-SARS-CoV-2 IgA and IgG antibodies was offered to HCWs at baseline and follow up two months thereafter in two hospitals of Brandenburg Medical School during the first wave of the COVID-19 pandemic in Germany in an ongoing observational cohort study. Medical history and signs and symptoms were recorded by questionnaires and analyzed. (3) Results: Baseline seroprevalence of anti-SARS-CoV-2 IgA was 11.7% and increased to 15% at follow up, whereas IgG seropositivity was 2.1% at baseline and 2.2% at follow up. The rate of asymptomatic seropositive cases was 39.5%. Symptoms were not associated with general seropositivity for anti-SARS-CoV-2; however, class switch from IgA to IgG was associated with increased symptom burden. (4) Conclusions: The seroprevalence of antibodies against SARS-CoV-2 was low in HCWs but higher compared to population data and increased over time. Screening for antibodies detected a significant proportion of seropositive participants cases without symptoms.

## 1. Introduction

Since its emergence in late 2019, more than 119,000,000 infections with the severe acute respiratory syndrome coronavirus-2 (SARS-CoV-2) have been diagnosed worldwide until December 2020. In addition, more than 2,650,000 deaths were observed with the new coronavirus disease 2019 (COVID-19) [1]. The infection has spread worldwide and has been categorized as a pandemic by the World Health Organization (WHO). In the United States of America, COVID-19 has recently been identified as the leading cause of death, especially for patients aged >45 years [2].

It has been recognized that SARS-CoV-2 causes a broad range of disease burden, from asymptomatic infection or mild clinical course to severe or even fatal illness. The majority of reports have focused on hospitalized patients representing a population with a more severe cause of the disease and thus only represent the tip of the iceberg. However, the vast majority of up to 80% of cases display a completely asymptomatic or only mild clinical course; data on these patients are scarce, yet several reports have suggested that even asymptomatic patients can infect others [3,4,5]. Furthermore, it has been proposed that the regional differences in mortality actually reflect the number of cases not revealed due to undiagnosed cases and differences in testing capacity, in particular in low-income countries [6].

However, healthcare workers (HCWs) and medical staff are at high risk for infection. This may lead to the paradox that those caring for COVID-19 patients may inadvertently contribute to spreading the disease if they are not readily sent into quarantine, reducing the much-needed capabilities of the healthcare system.

In Spain and Italy, where the initial wave of COVID-19 caused relevant morbidity in HCWs and patients, HCWs contributed to 15% and 9%, respectively, of positive PCR results for SARS-CoV-2 [7]. In the Italian outbreak in Lombardy, 20% of medical personnel became infected [8]. Overall, HCWs are seven times more likely to develop severe COVID-19 than other workers without professional contacts to patients, underlining the importance of that issue [9].

While PCR testing was available soon after isolation of the virus, serological assays for SARS-CoV-2-specific IgG, IgM and IgA have been established, but data are sparse. As antibodies are expected to be present for a longer time than the virus as detected by PCR and follow a typical sequence of IgA, IgM and IgG positivity [10], such assays may help in estimating cumulative infection rates and shed light on asymptomatic infections to learn more about the actual disease burden of COVID-19. COVID-19 serology testing can be performed at a higher throughput in less time and, given that analyses are performed in blood and not on nasopharyngeal swabs, is less dependent on sampling/specimen quality than PCR. Serial serological testing could thus be suitable to assess the prevalence of immunity and previous contact with SARS-CoV-2 in a population such as HCWs and to monitor the development of herd immunity.

Several serological assays for detecting SARS-CoV-2 have been approved by local authorities (e.g., the European Medicines Agency (EMA)) and have been published with specificity ranging from 85 to 99% [11] and may help in establishing or ruling out the diagnosis of COVID-19 [12]. In addition, diagnostic parameters of serological assays also depend on the time the blood samples were drawn relative to the onset of infection, as the sensitivity of the assays was shown to increase with time relative to symptom onset [13]. In a recent Cochrane analysis, pooled results for IgG, IgM, IgA, total antibodies and IgG/IgM all showed low sensitivity during the first week since the onset of symptoms (all less than 30.1%), rising in the second week and reaching their highest values in the third week, with pooled sensitivities of 96% three weeks after infection [14].

Recent serological data from Denmark suggest that HCWs have a higher rate of anti-SARS-CoV-2 seropositivity compared to blood donors [15]. In the general German population, more than 2,500,000 infections and >72,000 deaths in SARS-CoV-2-positive patients have been observed [16]. Seropositivity studies on HCW, however, have been scarcely reported from Germany. Recently published health insurance data from Germany suggest that HCWs are more than double as likely to suffer from COVID-19 than other occupational groups [17].

We report a prospective serological follow-up study of HCWs at two major university hospital sites of the Brandenburg Medical School in the rural federal state of Brandenburg, Germany. We took baseline and follow-up samples from healthcare workers during the first wave of the COVID-19 pandemic. The late arrival of this wave in these hospitals enabled us to take the baseline samples in the early phase of the first wave. Thus, this study allows for a population-based serological picture of HCWs from the early phase of the pandemic well into the ongoing second wave.

## 2. Materials and Methods

### 2.1. Situation before the Start of the Study in the Federal State of Brandenburg, Germany

On 30 March 2020, according to official data based on the Robert Koch Institute, the federal institute for infectious diseases of Germany, a cumulative quantity of 68,137 people tested positive for the SARS-CoV-2 genome in the federal state of Brandenburg (https://experience.arcgis.com/experience/478220a4c454480e823b17327b2bf1d4/page/page_0/ (accessed on the 14 February 2021). Taking into account the reported population of the federal state of Brandenburg of 2,523,087 people (https://www.statistik-berlin-brandenburg.de/ accessed on the 14 February 2021), this leads to an incidence/prevalence of SARS-CoV-2 positivity of approximately 2.7% in the general population at the time from which the first samples were available for this study.

### 2.2. Study Design and Enrolment

The Test-SARS-CoV-2-BB study is an ongoing bicentric exploratory healthcare worker cohort study in two centers of a medical school in the federal state of Brandenburg in Germany. Both hospitals have >500 beds and deliver healthcare for the local federal districts.

We here report the baseline characteristics at the time of initial sampling and the first follow-up assessment approximately 10 weeks later. Further follow-up assessments are planned. Study participation is voluntary, and consent can be withdrawn at any time. There are neither positive nor negative incentives to participate. The participants were enrolled in the two participating centers in Brandenburg/Havel and Neuruppin.

After informed consent, questionnaires were obtained, and blood samples were obtained. Testing was performed in the early phase of the first wave (in Brandenburg before the first known case was admitted to the hospital), thus representing a baseline screening prior to intensive exposure in the hospital setting.

Analyses were performed according to Good Clinical Practice (GCP) guidelines as well as in concordance with the Declaration of Helsinki. The study was approved by the local Ethics Committee (Ethics Committee of the Brandenburg Medical School “Theodor Fontane,” Brandenburg, Germany; “Tests zur Untersuchung von SARS-CoV-2 Inzidenz, Krankheitsverlauf und Prävalenz im Land Brandenburg (Test-SARS-CoV-2-BB)”; Aktenzeichen: E-01-20200409).

### 2.3. Questionnaire

The questionnaire was developed by the COVID-19 study group who are experienced internal medicine physicians. The goal of the questionnaire was to assess demographic data such as age and sex, job duties and locations, concomitant medication, medical history, travel history and possible COVID symptoms reported within the preceding 12 weeks according to the official guidelines by the Robert Koch Institute, the federal institute for infectious disease recommendations (https://www.rki.de/DE/Content/InfAZ/N/Neuartiges_Coronavirus/Falldefinition.html (accessed on 14 February 2021). Symptoms assessed were symptoms characteristic for COVID-19 such as fever, cough, dyspnea, loss of smell, dysgeusia and additionally unspecific but typical symptoms for virus infections such as myalgia, headache or fatigue.

### 2.4. Serological Testing

All analyses were performed according to the manufacturer’s recommendations (Euroimmun Medizinische Labordiagnostika, Luebeck AG, Germany).

Briefly, the ELISA microtiter plates used for this assay are coated with the recombinant S1 domain antigen of SARS-CoV-2. In the first step, diluted serum from HCWs or patients is incubated with the microtiter test tubes. If positive antibodies (IgA, IgG) are present in the serum sample they bind to the recombinant antigen. In the second step of the assay, peroxidase-linked anti-human-IgA or IgG reagent is incubated and thus used for the color reaction. The test results are determined from extinction values (OD = optical density). These OD values are used to calculate ratios by division of sample OD by calibrator OD. Subsequently, ratios determine the diagnostic findings. For IgA and IgG, a cutoff ratio <0.8 means a seronegative result, 0.8–1.1 means a borderline result and >1.1 a positive result. For IgA, the reported negative predictive value was between 0.975 and 100% and for IgG at 0.979–100%, respectively [13,18].

This assay previously demonstrated a diagnostic sensitivity of 95% and specificity of 96.2% for anti-SARS-CoV-2 IgG, as well as a specificity of 73.2% for anti-SARS-CoV-2 IgA, respectively [19]. Additionally, diagnostic performance has been shown to improve with days past onset of symptoms (PSO), as sensitivity increased from 71% PSO to 100% 14 days PSO for anti-SARS-CoV-2 IgA, while it was 63% after symptom onset and 100% 14 days after symptom onset for anti-SARS-CoV-2 IgG [13]. At the time of the initial study design and analyses, no IgM assay approved by the regulatory authorities in Europe was available; therefore, only anti-SARS-CoV-2 IgG and IgA were analyzed.

No concurrent SARS-CoV-2 PCR tests to evaluate active infection were obtained, as the availability of validated swabs and SARS-CoV-2 RT-PCR kits was scarce at that time and reserved for dedicated patient care.

### 2.5. Statistical Analysis

HCWs data were collected and inserted into a database (Microsoft Excel 2010; Microsoft Software, Redmond, WA, USA). Statistical analysis was performed with GraphPad Prism 5 (Graph Pad Software, La Jolla, CA, USA). The test for significance was performed with an unpaired Student’s *t*-test. The significance level was set at a two-sided *p*-value of 0.05.

## 3. Results

### 3.1. Characteristics of Healthcare Workers at Baseline

In total, 1013 HCWs from the two participating centers were included in the analysis. At baseline (timeframe from 30 March to 7 April 2020), clinical and demographic information, as well as blood samples, were retrospectively available for 1013 healthcare workers and included in the analysis. Of these, 87/1013 (=8.6%) HCWs were working in high-risk COVID units (COVID isolation ward, COVID intensive care unit). The median age of HCWs was 41 years (range: 19–65 years).

### 3.2. Prevalence of Anti-SARS-CoV-2 IgA and IgG at Baseline

At baseline, 119/1013 (=11.7%) HCWs tested positive for anti-SARS-CoV-2 IgA when using a cutoff of >0.8. When using the stricter cutoff of ≥1.1, 69/1013 (=6.8%) HCWs tested positive for anti-SARS-CoV-2 IgA. Only 21/1013 (=2.1%) HCWs were seropositive for anti-SARS-CoV-2 IgG with a cutoff of >0.8. Of these, 14/1014 (=1.4%) tested positive when the stricter cutoff of 1.1 was used.

### 3.3. Development of Anti-SARS-CoV-2 IgA and IgG in Follow-Up

Follow-up data were available for 858 HCWs; however, anti-SARS-CoV-2 IgG data were only available for 811 HCWs. The median time to the first follow up was 65 days.

The seroprevalence for anti-SARS-CoV-2 IgA increased to 129/855 HCWs (=15%), including the gray area cutoff of ≥0.8. When the stricter cutoff of 1.1 was used, seropositivity for anti-SARS-CoV-2 IgA was 10.4% (=89/855 HCWs). For IgG, we observed that 19/811 (=2.2%) HCWs became seropositive for anti-SARS-CoV-2 IgG with a cutoff of >0.8, and 14/811 (=1.6%) had a higher OD of >1.1.

**Figure 1:**
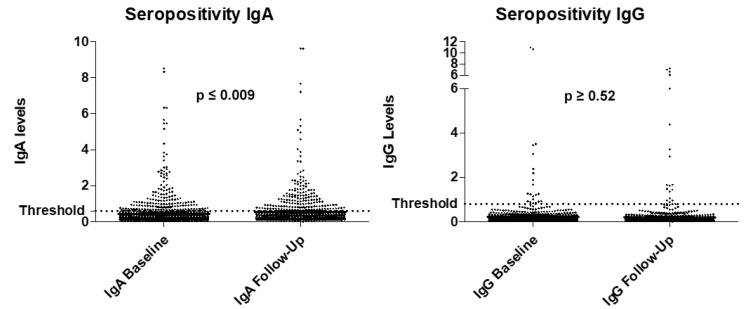
Levels of anti-SARS-CoV-2 IgA and anti-SARS-CoV-2 IgG at baseline and at follow up. Threshold positivity for positivity was set at 0.8 based on the manufacturers’ recommendations. The increase in seropositivity for anti-SARS-CoV-2 IgA was found to be statistically significant, while anti-SARS-CoV-2 IgG seropositivity was not.

The increase in anti-SARS-CoV-2 IgA positivity from 11.5 to 15% at follow up was found to be statistically significant (*p* ≤ 0.009). For anti-SARS-CoV-2 IgG, the increase in positivity was not significant (*p* ≥ 0.52). The frequency of positivity for anti-SARS-CoV-2 IgA and IgG is displayed in Figure 1.

### 3.4. Levels of IgA and IgG in Seropositive Healthcare Workers

At baseline, the median serological level of anti-SARS-CoV-2 IgA in seropositive HCWs was 1.22 (range: 0.8–8.5), while the level of anti-SARS-CoV-2 IgG was 1.25 (range: 0.84–10.99). At the time of the first follow-up, the median serological level of anti-SARS-Cov-2 antibodies was 1.23 (range: 0.8–6.3) for IgA and 2 (range: 0.89–7) for anti-SARS-CoV-2 IgG.

### 3.5. SARS-CoV-2 Seropositivity in COVID versus Non-COVID Wards

Positivity for anti-SARS-CoV-2 IgA was observed in 10/86 HCWs (11.6%) as opposed to 66/556 (=11.9%), suggesting that although a more frequent and intensive contact with COVID-positive patients was present, this did not lead to higher rates of infection. There was no significant difference in anti-SARS-CoV-2 IgA seropositivity between HCWs working in COVID versus non-COVID wards (*p* ≥ 0.95).

### 3.6. Low-IgG Response in SARS-CoV-2 IgA-Seropositive Participants

**Figure 2:**
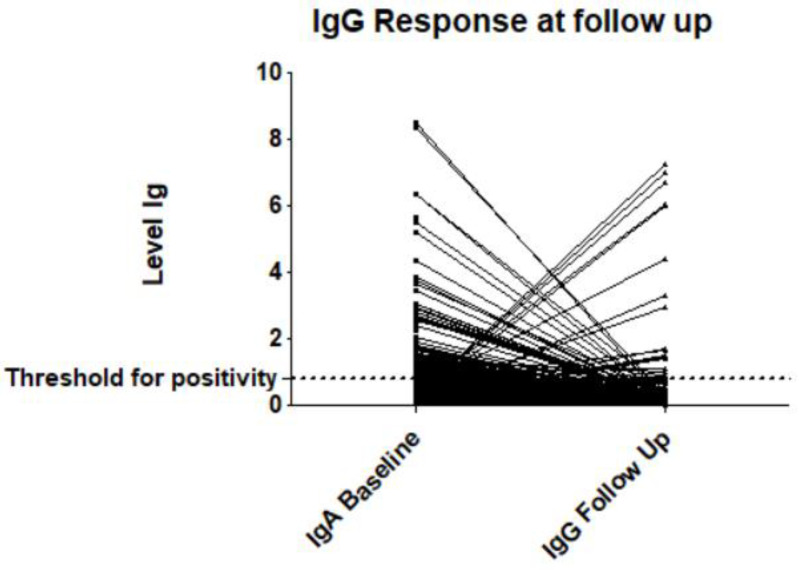
Levels of anti-SARS-CoV-2 IgA at baseline and anti-SARS-CoV-2 IgG at follow up. The threshold positivity for positivity was set at 0.8 based on the manufacturers’ recommendations.

Of those HCWs who tested positive for IgA at baseline, the individual course of IgG response at follow up was assessed. For 93/97 HCWs with IgA positivity at baseline, follow-up IgG data were available. Only 6/93 IgA-positive HCWs developed anti-SARS-CoV-2 IgG positivity at follow up (=6.5%). Another 13 HCWs showed anti-SARS-CoV-2 IgG positivity at follow up despite negativity for anti-SARS-CoV-2 IgA at baseline, suggesting an intercurrent contact with the virus. The course of anti-SARS-CoV-2 antibody levels (IgA at baseline to IgG at follow up) is displayed in Figure 2.

### 3.7. Symptom Burden/Frequency Relative to Anti-SARS-CoV-2 IgA Antibody Levels at Baseline

Symptom burden in HCWs relative to serologic antibody status was assessed. In HCWs who tested positive for anti-SARS-CoV-2 IgA, only 4/119 (=3.4%) cases reported fever, whereas 34/894 (=3.8%) seronegative HCWs experienced fever in the last 3 months. With regard to cough, 31/119 (=26%) IgA-seropositive HCWs reported cough, while 256/894 anti-SARS-CoV-2 IgA-negative HCWs (=28.9%) complained about having symptomatic cough in previous weeks. Myalgia, headache, fatigue and rhinitis were reported by 19/119 (=16.7%), 40/119 HCWs (=33.6%), 38/119 (=31.9%) and 30/119 (=25.2%) IgA-seropositive HCWs, while they were present in 119/894 (=13.4%), 364/894 (=40.7%), 312/894 (=34.9%) and 241/894 HCWs (=27%) who were found anti-SARS-CoV-2 IgA-negative, respectively. A total of 8 out of 119 (=6.7%) anti-SARS-CoV-2 IgA-seropositive HCWs reported dyspnea, while 71/894 (=7.9%) anti-SARS-CoV-2 IgA-negative HCWs had dyspnea. Only 5% (6 out of 119) of IgA-seropositive HCWs reported dysosmia or dysgeusia, while 3.8 % (34 out of 894) anti-SARS-CoV-2 IgA-negative HCWs reported having experienced that symptom in recent weeks.

There was no statistically significant difference between frequency or grading of reported symptoms or grading of symptoms between cases tested positive or negative for anti-SARS-CoV-2 IgA at baseline. The data and the corresponding *p*-values are depicted in Table 1.

Altogether, of those patients anti-SARS-CoV-2 IgA seropositive at baseline (*n* = 119), 47/119 (=39.5%) reported no symptoms at all in the past three months and were thus completely asymptomatic, although documented seropositivity suggested a previous, albeit unknown, exposure to SARS-CoV-2.

### 3.8. Symptom Burden Relative to IgG Response at Follow Up

As it could theoretically be assumed that cases showing an IgG response with developing anti-SARS-CoV-2 IgG might reflect a more intensive interaction with the virus and could therefore be accompanied by an intensified symptom burden or broader spectrum, a subgroup analysis of HCWs experiencing a class switch was performed.

For 811 HCWs, follow-up data for anti-SARS-CoV-2 antibodies were available. In order to evaluate if HCWs with documented seroconversion or class switch to SARS-IgG positivity at follow up (*n* = 19) had a more severe or distinct symptom burden, we evaluated symptoms in these 19 HCWs compared to HCWs who did not show an IgG response (*n* = 792).

Altogether, 19 out of 811 HCWs responded with IgG seropositivity of anti-SARS-CoV-2 IgG at follow up. Fever was reported in 2 out of 19 HCWs (10.5%) showing class switch versus 17 out of 792 (2.1%) HCWs being anti-SARS-CoV-2 IgG negative at follow up. IgA-seropositive HCWs with IgG response demonstrated cough in 15.8% (3 out of 19) versus 10.6% (17 out of 792) of HCWs without seroconversion. Aches of the limbs were reported in 4 out of 19 IgA-seropositive HCWs with IgG response (16.7%), while 71 out of 792 (9%) HCWs who did not display anti-SARS-CoV-2 IgG at follow up did report that symptom.

In HCWs showing anti-SARS-CoV-2 IgG seropositivity at follow up, headache was reported in 11 (57.9%), dyspnea in 6 (31.6%) and fatigue in 9 (47.4%) cases, while in HCWs without IgG responses, these symptoms were less frequent (30.9% for headache, 4.8% for dyspnea and 27% for fatigue). Dysosmia or dysgeusia was reported more frequently in IgA-seropositive HCWs showing an IgG response (15.8%) as opposed to those without (2%). Rhinitis was not found to be a discriminating symptom.

Symptoms statistically more frequent in IgA-seropositive cases developing an IgG response were fever, headache, dyspnea, fatigue and dysosmia/dysgeusia, with dyspnea and dysosmia/dysgeusia showing the highest statistical significance or discriminating ability.

Altogether, IgA-seropositive HCWs who demonstrated an IgG response were less often asymptomatic compared to those without class switch (26.3% versus 49.5%). The data and the corresponding *p*-values are displayed in Table 2.

## 4. Discussion

In this prospective and ongoing trial, we evaluated the serologic status of HCWs in the face of the ongoing COVID-19 pandemic in two large university hospitals of Brandenburg Medical School in the rural federal state of Brandenburg, Germany. As the first wave reached this region comparably late, it was possible to document seropositivity at an early epidemiologic stage of the pandemic, i.e., prior to admittance of the first COVID-19 patient, at least in Brandenburg/Havel.

At baseline, by the end of March 2020, “before the first Brandenburg wave,” we found a seropositivity rate in HCWs of 11.7% and 10.4% at optical density cutoffs of 0.8 and 1.1, respectively, which is broadly in keeping with other recent reports on seroprevalence in HCWs [20,21]. Seropositivity was then considerably higher than the published infection rate in the general population. At first glance, this may describe an actual difference in infections, which could be explained by a higher exposure of HCWs to SARS-CoV-2 compared to the general population.

Among those participants with positive SARS-CoV-2 serology, approximately 40% did not recall any symptoms consistent with a viral illness in the previous months. This suggests that a substantial number of SARS-CoV-2 infections in HCWs remain undetected, presumably due to the subclinical nature of some infections or underreporting of symptoms. In the follow-up serology analysis, a small yet statistically significant increase in anti-SARS-CoV-2 IgA positivity compared to baseline was found, while IgG was very similar to baseline. The observed increase in IgA positivity in the follow-up samples might either be due to an increase in sensitivity relative to the onset of infection [13] or the growing prevalence in the population. The persistently low prevalence of IgG positivity is in accordance with the very low number of new infections during the observed 2–3-month period between March/April and June 2020 in the northeastern parts of Germany.

We did not see a significant difference in seropositivity rates between participants working in COVID wards compared to non-COVID wards, arguing against nosocomial infections and shifting the focus on ambulant infections, suggesting that rapidly established, thorough precautions and safety measures (e.g., isolation, single room, personal protective equipment (PPE)) were effective at preventing SARS-CoV-2 exposure in these high-risk areas [22]. Indeed, data from earlier studies suggest that appropriate use of PPE reduces the spread and infection rates of airborne viral particles such as influenza and coronavirus [23]. Recent data during the COVID-19-pandemic seem to confirm this finding [24,25,26] and suggest that HCWs consistently using intensive PPE (N95 masks, eye protection) present more often as asymptomatic when tested positive for SARS-CoV-2 [27], presumably reflecting lower infectious load.

When analyzing symptom burden in depth, comparing HCWs seropositive for SARS-CoV-2 with those seronegative revealed no difference between these groups, which underscores the finding that a large proportion of seropositive HCWs present asymptomatic. Other studies show rather variable data regarding symptoms. Among patients with COVID-19, asymptomatic infections have been reported in 7.9 to 87.8% [28], and this is probably influenced by sample size and maybe the clinical setting (outpatient versus hospitalized).

Among the limited data available for HCWs, the study by Self et al. on a USA multistate hospital network found that 29.1% of seropositive HCWs recalled no symptoms, while contrary to our findings, their overall symptom burden was higher than in seronegative HCWs (71% versus 43%, respectively) [20].

One reason for this finding may be that the authors employed a pan-immunoglobulin assay that detects anti-SARS-CoV-2 immunoglobulin regardless of its isotype, thus not distinguishing between IgM, IgG and IgA. In our study, we did not find a statistically significant difference in symptom burden between HCWs who tested positive versus negative for IgA antibodies at baseline. Extending our analysis to all seropositive HCWs at baseline regardless of immunoglobulin class, we found 141 (12.7%) positive for either IgA, IgG or both, but did not observe significantly more symptoms in seropositive patients. In theory, this could be attributed to the possibly lower specificity of anti-SARS-CoV-2 IgA as compared to IgG, which might lead to false-positive results. According to the manufacturer’s leaflet, specificity of the EUROIMMUN IgA ELISA was 98.3% in samples obtained from the pre-COVID era [29] and additionally did not show cross-reactivity to other coronaviruses [30], which would argue against the majority being false-positive samples. Nilsson et al. recently recommended increasing the cutoff for anti-SARS-CoV-2 IgA to a 4.0 ratio, as this increased the specificity to >97% in their work [19]. Therefore, when we reanalyzed the IgA-seropositive subgroup of HCWs with a cutoff of ≥4.0 OD at baseline (*n* = 10) and compared them to those with a cutoff <0.8 OD (=negative), we still could not detect an increased symptom burden or spectrum in the group with higher seropositivity (data not shown).

Asymptomatic COVID-19 patients have been shown to have lower levels of anti-SARS-CoV-2-specific CD4+T cell responses and specific IgA, IgG and IgM antibodies against SARS-CoV-2 [31]. This could explain the lack of differences observed, as nearly half of the IgA-seropositive participants in our study reported no symptoms at all. In contrast, HCWs displaying a direct IgG response or a “class switch” experienced symptoms of viral upper airway disease such as dyspnea, fever, fatigue or dysosmia/dysgeusia significantly more often. From a pathophysiological point of view, this could hint at an intensified immunological interaction with the virus, possibly conferring a higher symptom load. This phenomenon has previously been described for patients directly converting from seronegativity to IgG seropositivity [32]. Whether the same holds true for cases of baseline IgA seropositivity responding with IgG has not been published but appears to be likely. Interestingly, it has been suggested that asymptomatic individuals had a weaker immune response to SARS-CoV-2 infection and lower levels of anti-SARS-CoV-2 IgG [33], which supports our finding.

As another interesting finding, a small proportion (*n* = 13 out of 89) of HCWs who tested positive for IgA at baseline fell below the threshold of 0.8 OD in the follow-up analysis. Apart from false-positive IgA tests, this may reflect patients whose antibody levels actually declined and was observed particularly for IgA [34].

Our study has several limitations that need to be addressed. (a) As samples were obtained in a scientific study setting, no concurrent SARS-CoV-2 PCR tests to evaluate active infection were obtained, as availability of validated swabs and SARS-CoV-2 RT-PCR kits was scarce at that time and reserved for dedicated patient care [35]. This prohibited us from calculating diagnostic parameters such as sensitivity and specificity of the assay. Additionally, comparisons of incidence between HCWs and the general population can only be inflected from the positivity rate among HCWs in the participating hospitals, not from the exact study population. There are good reasons to assume that both samples, participants in our study and HCWs tested by PCR, are representative of the local HCW population, but we cannot prove this. (b) The number of positive cases in total is rather small, which might impact the statistical validity. However, this is due to the small case numbers/incidences during the first wave of the pandemic, and continuation of our study may reveal patterns from the early phase through to the second wave of the pandemic. (c) Seroprevalence may be underestimated if infected HCWs had not yet developed an antibody response or even if antibody titers had declined since infection.

In summary, we could demonstrate that HCWs show substantial anti-SARS-CoV-2 seropositivity, even if no symptoms were present. This might indicate the high number of asymptomatic and therefore unrecognized cases. Additionally, we observed that “seroconversion” to or response with IgG is associated with symptomatic disease and increased symptom burden. As it is currently unknown whether asymptomatic carriers pose an infection threat to others, serological analysis may thus be a useful method in evaluating the current prevalence and may help reduce the further spread of SARS-CoV-2, thus supporting the safety of HCWs and their patients. Additionally, especially in those countries with a low prevalence of COVID-19 and thus a low pretest probability, serological analyses for anti-SARS-CoV-2 might be a good complementary test for patients to increase the diagnostic accuracy by confirming the diagnosis or ruling out a false-positive result [12].

We also found that a considerable proportion of seropositive participants tend to “lose” their seropositivity. Whether this goes along with a loss of immunity or rather points at the predominant biological role of cellular immunity remains to be investigated. It appears, though, that cumulative long-term prevalence will not be possible to be estimated on the basis of serological testing. The study is currently ongoing, and follow-up samples will be analyzed.

## 5. Conclusions

In HCWs, the seroprevalence of antibodies against SARS-CoV-2 was low but higher compared to population data from public registries and increased over time. The screening for antibodies in HCWs detected a significant proportion of asymptomatic cases, suggesting the need for screening procedures based on RT-PCR or lateral-flow devices as well as serological tests, as it is currently unclear if asymptomatic carriers may represent a potential transmission to patients or coworkers.

## Figures and Tables

**Table 1:** Symptom spectrum and significance in HCWs relative to Anti-SARS-CoV-2-IgA serostatus.

Symptom	Anti-SARS-CoV-2-IgA Positive (*n* = 119) ^#^	Anti-SARS-CoV-2-IgA Negative (*n* = 894) ^#^	*p*-Value *	Highest Grading IgA Positive ^£^	Highest Grading IgA Negative ^£^
Fever	4 (3.4%)	34 (3.8%)	≥0.83	II	II
Cough	31 (26%)	256 (28.9%)	≥0.60	III	III
myalgia	19 (16.7%)	119 (13.4%)	≥0.40	III	III
Headache	40 (33.6%)	364 (40.7%)	≥0.15	III	III
Dyspnea	8 (6.7%)	71 (7.9%)	≥0.66	III	III
Fatigue	38 (31.9%)	312 (34.9%)	≥0.57	III	III
Rhinitis	30 (25.2%)	241 (27%)	≥0.73	n.a	n.a
Dysosmia/dysgeusia	6 (5%)	34 (3.8%)	≥0.51	II	II
No symptoms	47 (39.5)	38 (4.3%)			

# Health care workers (HCW) with blood samples with missing questionnaire were left out of the analysis; * probability value of the exact Fisher test (*p*-value); ^£^ According to grading by Common Toxicity criteria 4.0.

**Table 2:** Symptom spectrum and significance in HCWs relative to Anti-SARS-CoV-2-IgG class switch/seroconversion.

Symptom	HCWs Anti-SARS-CoV-2- IgG Positive (Class Switch Positive) (*n* = 19)	HCWs Anti-SARS-CoV-2- IgG Negative (Class Switch Negative) (*n* = 792)	*p*-Value ^¥^
Fever	2 (10.5%)	17 (2.1%)	≤0.015 *
Cough	3 (15.8%)	84 (10.6%)	≥0.447
myalgia	4 (21.1%)	71 (9.0%)	≥0.064
Headache	11 (57.9%)	245 (30.9%)	≤0.009 *
Dyspnea	6 (31.6%)	38 (4.8%)	≤0.0001 *
Fatigue	9 (47.4%)	214 (27%)	≤0.039 *
Rhinitis	3 (15.8%)	95 (12%)	≥0.61
Dysosmia / dysgeusia	3 (15.8%)	16 (2%)	≤0.0001 *
No symptoms	5 (26.3%)	392 (49.5%)	≤0.044 *

¥ probability value of the one-sided *t*-test; * statistically significant.

## Data Availability

The data presented in this study are only available on request from the corresponding author. The data are not publicly available due to privacy reasons as they contain sensitive HCW data.
